# Awareness of retinopathy of prematurity among pediatricians in West Bank, Palestine: a descriptive study

**DOI:** 10.1186/s12886-018-0876-1

**Published:** 2018-08-13

**Authors:** Mohammad T. Akkawi, Jamal A. S. Qaddumi, Hala R. M. Issa, Liana J. K. Yaseen

**Affiliations:** 1 Department of Special Surgeries, Faculty of Medicine and Health Sciences, An-Najah National University Hospital, An-Najah National University, Nablus, Palestine; 2 An-Najah National University, Nablus, Palestine

**Keywords:** Awareness, Pediatricians, Retinopathy of prematurity

## Abstract

**Background:**

Retinopathy of prematurity (ROP) is a disorder of the developing retina of preterm infants due to defective vasculogenesis. The aim of the study was to analyze the level of awareness, knowledge, attitude and practice of pediatricians about ROP in the West Bank, Palestine.

**Methods:**

A questionnaire was designed on the knowledge, attitude, and practice (KAP) pattern. The questionnaire included questions about pediatrician’s educational and practicing profile, knowledge of screening guidelines, risk factors for ROP, referral facilities and barriers for referral. The questionnaire was given to70 practicing specialists and residents in hospitals having neonatal intensive care units in the West Bank, Palestine. It was a self-administered questionnaire, collected between November 2016 and February 2017.

**Results:**

A total of 70 pediatricians from 11 different hospitals without ROP screening service participated in the study. The mean age of the participants was 33.04 ± 7.74. Of which, 62.9% were males and 37.1% were females. Fifty-nine (84.3%) answered that ROP is preventable, while 11 (15.7%) responded that ROP is not preventable. Nine (12.9%) pediatricians had no idea as to which part of the eye is affected in ROP. Among the participants, 29 (41.4%) did not know when ROP screening should be started. Sixty-three (90%) pediatricians were sure that ROP is treatable. Regarding barriers for ROP screening, ‘ophthalmologist not available’ reason was expressed by 37.1% (26/70), ‘discharge person not writing’ by 20% (14/70) and ‘parents not agreeing’ by 18.6% (13/70) of the participants. Knowledge on the use of laser as a treatment modality of ROP was shown by 39 (55.7%) participants, and the use of anti-VEGF was shown by 6 (8.6%) participants, whereas 25 (35.7%) of the participants didn’t know about the treatment modalities.

**Conclusion:**

The study findings suggest that a large majority of pediatricians were aware of ROP as a preventable disease, but had less information about ROP screening guidelines and service delivery. The study suggests the need to increase the awareness of pediatricians by dissemination of information about ROP and creating a close coordination between them and ophthalmologists to address barriers for service delivery in Palestine.

## Background

Retinopathy of prematurity (ROP), previously called retrolental fibroplasias, is a vasoproliferative disorder of the retina which occurs principally in premature children due to defective vasculogenesis as a result of exposure to risk factors [1]. It mainly affects premature babies born at or before 32 weeks and weighing 1500 g or less at birth [2]. However, larger and more mature infants can develop severe ROP in countries with low/ moderate levels of development compared with highly developed countries [3]. Several risk factors have been found to be associated with the development of ROP. The most identified are low gestational age, low birth weight, poor postnatal growth, inadequately administered oxygen supplementation, respiratory distress and blood transfusion. Most studies show the most significant factors to be low gestational age and low birth weight [4, 5]. A significant improvement of the standards in neonatal intensive care units (NICUs) and perinatal care has increased the survival rate for the premature babies over the last decades. Consequently, the incidence of ROP has increased in parallel [6].

Retinal examination in preterm infants should be performed by an ophthalmologist with a sufficient knowledge and experience in order to identify accurately the location and sequential retinal changes of ROP [7]: Screening would be undertaken in the neonatal unit for babies who are still in-patients. Discharged babies can be examined in the neonatal unit during follow up, or in eye departments. Revised guidelines for screening have been suggested by the American Academy of Pediatrics, the American Association for Pediatric Ophthalmology and Strabismus and the American Academy of Ophthalmology. Those recommend screening for ROP in all infants with birth weight < 1500 g or gestational age of 32 weeks or less [8]. In contrast, the British Association for Perinatal Medicine and the College of Ophthalmologists recommend screening only infants < 1500 g at birth [9]. Timing of the initial examination is based on both postmenstrual age (PMA) and chronological age (CA) and is undertaken to detect 99% of infants at risk of a poor visual outcome. The first examination is conducted between four and nine weeks CA, depending on PMA at birth. Subsequent studies have confirmed the efficacy of conducting the first examination at four weeks CA in more mature infants [10]. These criteria only apply to these high income settings, wider criteria are used in less well resourced settings and less developed countries to ensure that all babies at risk are examined [3].

The current gold standard in treating sever, Type 1 ROP is still panretinal laser photocoagulation. However, ongoing studies in premature infants have been investigating the safety and efficacy of antiangiogenic therapies, especially anti-VEGF drugs. This seems to provide valuable and encouraging information for ROP treatment in the near future, though the long-term ocular and systematic safety of Anti-VEGF agents is not yet known [11]. A study was done in South India showed that awareness of ROP is poor among pediatricians. The same study mentioned that reports from other developing countries like China, Thailand and Vietnam also show a similar trend [12]. A study was done in 2013 in Nigeria to determine the level of awareness of the screening protocols for ROP among pediatricians, concluded that although majority of pediatricians are aware of ROP, they are poorly informed on the management and screening of the condition, and they need to be educated to be aware in order to prevent this treatable cause of blindness in children [13]. Another study was conducted in India 2011 to evaluate the prevailing practices for screening and referral scheme among Indian pediatricians for ROP concluded that only 14.5% were following international recommendations for ROP referral and that the screening remains poor due to the non-availability of trained ophthalmologists as well as inconsistent screening guidelines [14].

Retinopathy of prematurity is emerging as an important cause of avoidable blindness in both developed and developing countries [15]. Proper screening and referral practices will help saving sight for a more productive life. This is vital as number of blind years in a child increases the burden of care upon the family affected and the society as a whole [16, 17]. To the best of our knowledge, no studies were done in Palestine concerning ROP screening or awareness among pediatricians. This study was conducted to assess knowledge, attitude and practice patterns (KAP) of pediatricians about ROP.

## Methods

The study was a cross-sectional non-interventional descriptive study. It was conducted in eleven different Palestinian hospitals (private and governmental) in the West Bank that contain neonatal intensive care units (NICUs) and have no ophthalmology departments. Pediatricians usually consult a specialized ophthalmologist from outside to screen cases with high risk for ROP, any intervention was undergone in a specialized hospital. We invited seventy pediatricians (specialists and residents) who are in direct contact with neonatal care who all participated in the study. The data were collected between November 2016 and February 2017. A self-administered semi-structured questionnaire was formed on the knowledge, attitude and practice (KAP) pattern. It was gathered and modified from other questionnaires of similar published researches to assess knowledge, screening, referral barriers and treatment of ROP [12].

The study was approved by An-Najah National University IRB committee. Also, informed consent was taken from each participant, explained clearly that participants’ privacy was insured.

## Results

Seventy pediatricians participated in the study. The demographic details are given in Table 1. The mean age of the participants was 33.04 + − 7.74 years, range 25 to 59 years. 44 (62.5%) were males. There were 31 (44.3%) qualified pediatricians, while residents in training were 39 (55.7%). 16 (22.6%) had been practicing for more than 10 years, 14 (19.9%) had about 5 to 10 years of practice experience and 40 (57.2%) had less than 5 years practice experience. Most of the respondents, 67(95.7%) were governmentally employed.

**Table 1:** Demographic details of the participants

Variable	Category	Frequency	Percent
Gender	Male	44	62.5
	Female	26	37.1
Age (years)	25–35	48	68.6
	36–45	16	22.9
	46–55	5	7.1
	56–65	1	1.4
Educational qualification	Specialist	31	44.3
	Resident	39	55.7

Fifty nine (84.3%) pediatricians said that ROP is preventable, 3 (4.3%) responded that ROP is not preventable, and 8 (11.4%) did not know. Twenty-four (34.3%) participants thought that blindness due to ROP is reversible, 35 (50.0%) said it is irreversible, whereas 11 (15.7%) did not know (Table 2).

**Table 2:** Awareness of participants regarding risk factors of retinopathy of prematurity and who performs screening for retinopathy of prematurity

Variable	Category	Frequency	Percent
Causes of ROP	Low gestational age	2	2.9
	Weight less than 1800 g	5	7.1
	Sick require oxygen	2	2.9
	All the above	60	85.7
	Don’t know	1	1.4
Eye test to be performed By	Retina specialist	21	30.0
	Pediatric ophthalmologist	48	68.6
	Don’t know	1	1.4

Regarding ROP identification, 61 (87.1%) mentioned that it is identified by examining the retina and 9 (12.9%) did not know. Table 2 shows pediatrician’s awareness about who performs screening for ROP.

Table 3 shows percentages of pediatricians with respect to their idea on the period of first eye test for ROP screening, that is, when to refer to ophthalmologist for ROP screening.

**Table 3:** Awareness of participants regarding timing of ROP screening

Variable	Category	Frequency	Percent
Period for first eye test	4–6 weeks	41	58.6
	Depend on the gestational age	25	35.7
	Don’t know	3	4.3
	Other	1	1.4

Referral for ROP screening was routinely undertaken by 67 (95.7%) respondents. The major two methods of referral among those were writing a discharge slip (60%) or informing the parents verbally (42.8%). The main reason given for not screening for ROP was the unavailability of an ophthalmologist in the same hospital among 26 (37.1%). Other contributing reason had to do with the discharging doctor not writing a screening referral order in 14 participants (20%).

Sixty-three (90.0%) pediatricians were sure that ROP is treatable, while 7 (10.0%) thought the opposite. Knowledge on the use of laser or anti-VEGF injection as a treatment modality of ROP was recorded in 39 (55.7%) and 6 (8.6%) participants respectively. 22 (31.4%) did not know how ROP is treated. In response to the question regarding the participants’ opinion on how successful the treatment of ROP is in preventing blindness, only 26 (37.1%) thought it is good, 17 (24.3%) said very good, 26 (37.1%) considered it satisfactory, while only one (1.4%) thought it is poor.

Only 16 (22.9%) pediatricians were satisfied with their current status of awareness and knowledge on ROP, while 30 (42.9%) were not satisfied, 24 (34.3%) had no idea about the current status on the awareness and knowledge.

## Discussion

Retinopathy of prematurity, when severe can result in permanent visual disability, causing a high financial burden for the community and the individual. Guidelines have been established in some countries to enhance early identification and prompt treatment of babies with ROP. Pediatricians who are the primary caregivers of premature babies ought to be aware of the risk factors, screening and referral protocols, as well as treatment modalities and outcomes. This will lead to improvement in the quality of care of these babies, and thus saving their sight, along with the inherent costs of blindness to the individual and community [13].

In our study, although the majority of pediatricians had knowledge about some of the risk factors for ROP, a significant number of them were not aware of the correct timing of ROP screening. This may be due to the lack of clear established screening protocols for ROP in Palestine, and so it may contribute to a delayed detection of the problem.

The American Academy of ophthalmology clarified that indications for screening are region dependent. Thus, it is advisable to develop more researches about possible risk factors and screening criteria specific to country, instead of relaying merely on the criteria published by developed countries. Gilbert [3] advised those who did not have local screening guidelines for ROP to use wide screening criteria.

Identifying barriers for referral is vital for formulating any successful model. In our study the major reason given for not screening for ROP was the unavailability of an ophthalmologist in the same hospital according to over a third of the participants. In a study done by Mohammad et al. from the United States about barriers for ROP screening or follow-up eye care after discharge from the NICU, it was found that infants who were not screened for ROP in the NICU had a greater risk for missing follow-up eye care compared to those who had their first retinal examination in the NICU [18]. This highlights the importance of screening taking place in the NICU and disseminating information about ROP in our hospitals.

Unfortunately, some of the participants in our study were sure that ROP is not treatable. This may affect their willing to refer cases for screening, and thus prevents early detection and treatment of such a preventable issue.

The role of the nursing staff is critical to a successful prevention of ROP- induced blindness as they help in monitoring oxygen saturation targets during the whole period of treatment. They are responsible for feeding and temperature control and play a vitally important role in preventing late sepsis, they participate actively in the recommendations to the patient’s family regarding follow up examinations after discharge [19].Apart from this, many cases of prematurity are babies of multiple gestations after IVF as their parents complained of infertility for years, so there might be a need to raise awareness among obstetricians and parents who are contemplating IVF as well.

In our study, only 16 (22.9%) pediatricians were satisfied with their current status on awareness and knowledge on ROP. Up to now, there are no studies concerning KAP for ROP among pediatricians published in Palestine. We hope this study to be a step in enhancing knowledge among pediatricians, as well as establishing clear protocols concerning referral and screening of ROP.

Our study had some limitations, as with all self-reported questionnaires, the data could not be validated as their practices were not observed. It did not include other health care providers i.e. nurses in the NICU, Also a question about the place where the babies are usually screened was not included in the questionnaire.

In order to improve the awareness of ROP among pediatricians, nurses, obstetricians, and parents we recommend appropriate coordination between pediatricians and ophthalmologists, as well as dissemination of information through publishing articles and seminars in medical conferences, medical journals and health education materials for parents so local multidisciplinary workshops to highlight this issue is recommended.

## Conclusion

The study findings suggest that a large majority of specialists and residents were aware of ROP as a disease, but had less information about ROP screening guidelines, service delivery and treatment modalities. The study suggests the need to create close coordination between pediatricians and ophthalmologists to address screening guidelines and barriers for service delivery in our country. The vital role of pediatricians in any screening program supports the need to enlighten them and increase their awareness by dissemination of information about ROP through seminars and literature, as well as spreading awareness among key members involved in preterm baby care.

## Abbreviations

CA: Chronological age
GA: Gestational age
KAP: Knowledge, attitude, and practice
NICU: Neonatal intensive care unit
PMA: Postmenstrual age
ROP: Retinopathy of prematurity
